# Genetic link between renal birth defects and congenital heart disease

**DOI:** 10.1038/ncomms11103

**Published:** 2016-03-22

**Authors:** Jovenal T. San Agustin, Nikolai Klena, Kristi Granath, Ashok Panigrahy, Eileen Stewart, William Devine, Lara Strittmatter, Julie A. Jonassen, Xiaoqin Liu, Cecilia W. Lo, Gregory J. Pazour

**Affiliations:** 1 Program in Molecular Medicine, University of Massachusetts Medical School, Biotech II, Suite 213 373 Plantation Street Worcester, Massachusetts 01605, USA; 2 Department of Developmental Biology, University of Pittsburgh, 8111 Rangos Research Center, 530 45th Street, Pittsburgh, Pennsylvania 15201, USA; 3 Children's Hospital of Pittsburgh, University of Pittsburgh Medical Center, Children's Hospital Drive 45th Street and Penn Avenue Pittsburgh, Pennsylvania 15201, USA; 4 Electron Microscopy Core, University of Massachusetts Medical School, 55 Lake Avenue North, Worcester, Massachusetts 01655, USA; 5 Department of Microbiology and Physiological Systems, University of Massachusetts Medical School, 55 Lake Avenue North, Worcester, Massachusetts 01655, USA

## Abstract

Structural birth defects in the kidney and urinary tract are observed in 0.5% of live births and are a major cause of end-stage renal disease, but their genetic aetiology is not well understood. Here we analyse 135 lines of mice identified in large-scale mouse mutagenesis screen and show that 29% of mutations causing congenital heart disease (CHD) also cause renal anomalies. The renal anomalies included duplex and multiplex kidneys, renal agenesis, hydronephrosis and cystic kidney disease. To assess the clinical relevance of these findings, we examined patients with CHD and observed a 30% co-occurrence of renal anomalies of a similar spectrum. Together, these findings demonstrate a common shared genetic aetiology for CHD and renal anomalies, indicating that CHD patients are at increased risk for complications from renal anomalies. This collection of mutant mouse models provides a resource for further studies to elucidate the developmental link between renal anomalies and CHD.

## Abstract (web summary)

Using forward genetic screen in fetal mice, Gregory Pazour and colleagues describe mutants affecting kidney/urinary tract development. The authors also show that mutants that cause kidney defects overlaps with those leading to congenital heart defects, thus linking renal anomalies and congenital heart disease.

## Graphical abstract

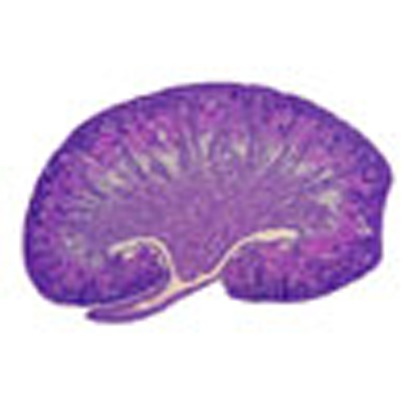

Developmental abnormalities of the kidney account for 20–30% of all structural birth defects and are a significant cause of end-stage renal disease^1^,^2^,^3^. Development of the metanephric or adult kidney in mammals starts with the formation of the ureteric bud from the mesonephric duct. Development is driven by reciprocal interactions between the epithelium of the mesonephric duct and the metanephric mesenchyme that overlies the nephric duct. These interactions induce the ureteric bud to grow out, branch and differentiate into the collecting ducts, the renal calyces, pelvis and ureter. The metanephric mesenchyme is reciprocally induced to undergo a mesenchyme-to-epithelial transition to form the nephrons.

Defects in the induction and patterning of the developing kidney can lead to a variety of renal-related birth defects that collectively are known as congenital abnormalities of the kidney and urinary tract (CAKUT). These can include renal/urinary tract-specific abnormalities, kidney agenesis (absent kidney), hypoplasia (small kidney) or dysplasia (immature or malformed structures in the kidney), cystic dysplastic kidneys (malformed kidneys with cysts), horseshoe kidney (both kidneys fused together), hydronephrosis (dilation of the renal pelvis), hydroureter (dilation of the ureter), duplex kidneys (duplicate collecting duct systems with one branched or two unbranched ureters), as well as obstructions at the ureter–bladder junction or ureter–kidney pelvis junction. Renal defects can also be observed as part of syndromes such as in VACTERL association, where they are seen in combination with vertebral defects, anal atresia, cardiac defects, trachea–oesophageal fistula and limb abnormalities^4^. In renal coloboma syndrome, defects in formation of the eye and kidney are observed, whereas Fraser syndrome affects formation of the kidney and genitalia. In addition, ciliopathies often present with cystic kidney disease in combination with other organ defects.

The genetics of the major forms of cystic kidney disease are well established with two genes (*PKD1* and *PKD2*) identified to cause autosomal dominant form of polycystic kidney disease (PKD) and a single gene (*PKHD1*) causing the recessive form of PKD. In addition, many genes causing renal ciliopathies have been identified. These typically encode components of the cilium or the machinery used to assemble cilia. However, the genetics for the majority of CAKUT cases remain largely unknown. The CAKUT genes that have been identified are typically involved in the early steps of patterning and development of the kidney. Recovery of CAKUT genes has been confounded by incomplete penetrance and the probable involvement of more complex genetics. Other than *Pax2*, which causes renal coloboma syndrome, and *Hnf1b*, which causes renal cystic disease and diabetes, most genes identified to cause CAKUT are associated with disease in only a few patients and the evidence supporting pathogenesis is weak^2^. In the present study, we examined renal anomalies in mutants recovered from a large-scale mouse mutagenesis screen. These mutants were identified to have congenital heart disease (CHD) and were recovered based on cardiovascular phenotyping using non-invasive fetal ultrasound imaging^5^. This allowed recovery of mutants that otherwise would be lost due to prenatal/neonatal lethality. Interestingly, many of the CHD mutants recovered also exhibited renal abnormalities. The recovery of pathogenic mutations in these mutants identified an enrichment of cilia-related genes, suggesting ciliary dysfunction may play a significant role in congenital defects of both the heart and kidney.

## Results

### Forward genetic screen identifies diverse kidney anomalies

We conducted a large-scale forward genetic screen with ethylnitrosourea mutagenesis, to interrogate the genetic aetiology of CHD^5^. With the screening of more than 80,000 fetuses using *in utero* ultrasound imaging, >200 mutant lines with a wide spectrum of CHD were recovered. The causative mutations were identified in 135 lines by whole exome sequencing and genotyping. As part of this study, we also characterized the non-cardiac anomalies in the CHD mutants and found an unexpected high prevalence of renal abnormalities in the CHD mutants. Among 135 lines in which the pathogenic mutations were recovered, we observed 39 had kidney defects (Table 1 and Supplementary Tables 1 and 2). The 39 lines had mutations in 11 genes that are cilia related and 15 genes that are not known to be cilia related (Table 1).

**Figure 1:**
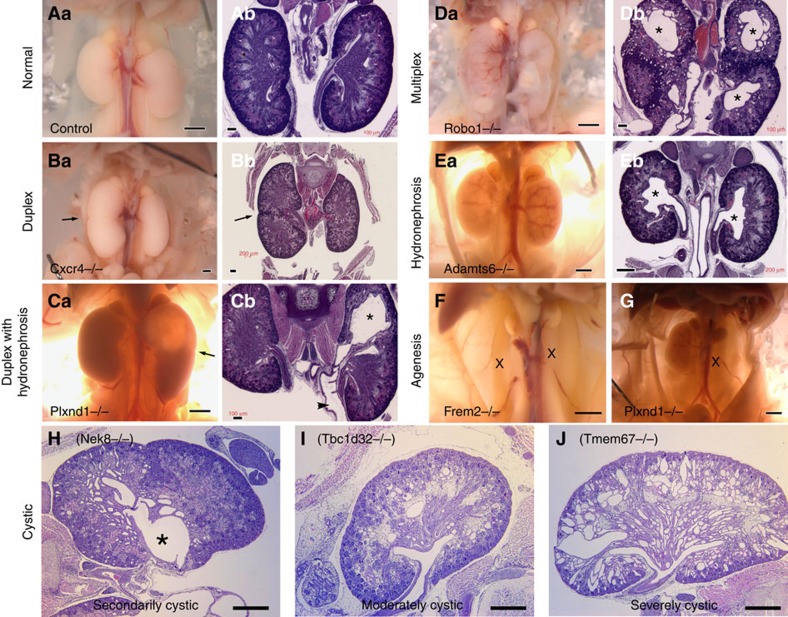
Phenotypes observed in screen. (**A**–**G**) Representative examples of the types of kidney defects observed. The **a** (left) image of most pairs is a necropsy image and the **b** (right) image is a haematoxylin and eosin (H&E)-stained image. Arrows point to the indentation that typically marks the junction between the two parts of a duplex kidney (**Ba**,**Bb**,**Ca**). Duplex kidney is thought to result from extra ureteric buds forming at the earliest steps of metanephric kidney development. The extra ureteric buds induce the formation of multiple (usually two) partial kidneys within a single capsule. If the second ureter does not properly drain, hydroureter (**Cb**, arrow head) and hydronephrosis (**Cb**,Eb*) can result. It is noteworthy that multiplex kidneys are similar to duplex kidneys, except that more than two kidneys are fused together on each side of the animal (**Da**,**Db**). The X marks the position of the missing kidneys in the agenesis images (**F**,**G**). Scale bars, 1 mm (**Aa**,**Ba**,**Ca**,**Da**,**Ea**,**F**,**G**). Scale bars, 100 μm (**Ab**,**Cb**,**Db**) and 200 μm (**Bb**,**Eb)**. (**H**–**J**) Bottom row shows examples of the different types of cystic kidney disease identified in the ENU screen. It is noteworthy that the Nek8 kidney is duplex and only the hydronephrotic (*) half of the kidney is cystic. This indicates that the cysts are secondary to the hydronephrosis. Moderate cystic disease was defined as a few large cysts accompanied by smaller dilations in the other tubes, while severe cystic disease was defined by having large cysts throughout the kidney. Scale bars, 500 μm (**H**–**J**).

Among the renal anomalies observed, the most common phenotype observed was duplex kidney, clinically known as duplicated collecting duct (Fig. 1b, *Cxcr4* and 1C, *Plxnd1*). The penetrance was variable, ranging from 15% in *Cep290* mutants to 66% in *Snx17* mutants (Table 1 and Supplementary Tables 1). Often the duplex kidneys were hydronephrotic, suggesting ureteral obstruction (Fig. 1c, *Plxnd1*). Duplex kidneys can arise from mutations in genes regulating ureteric bud formation on the mesonephric duct at the start of metanephric kidney development^6^. Duplex kidney has not been previously reported to be part of the ciliopathy spectrum and a role for cilia in ureteric bud formation is not currently known. However, our finding that four of the genes associated with duplex kidney are cilia related suggests that cilia are directly or indirectly regulating bud formation on the mesonephric duct. Hydronephrosis in the absence of duplex kidney was also observed with variable penetrance (Fig. 1e, *Adamts6*). Of the genes identified in this screen to cause duplex kidneys or hydronephrosis, only *Wnt5a* was previously shown to cause renal–urinary tract anomalies in humans. In humans, defects in *Wnt5a* cause Robinow syndrome, which includes skeletal, facial and genital abnormalities along with kidney and other anomalies^7^.

We also observed multiplex kidneys. This was associated with mutations in two non-cilia genes, *Robo1* and *Slit2*. These mutants developed bilateral multiplex kidneys (Fig. 1d, *Robo1*). *Robo2* and *Slit2* mutations were previously shown to cause the multiplex kidney phenotype by allowing many ureteric buds to form on the mesonephric duct^8^. Interestingly, our mutant (line 872) harbours a predicted pathogenic mutation in the *Robo2* orthologue *Robo1*. In addition, a second potential splice-site mutation was observed in *Robo2*, but transcript analysis of the skin tissue showed no *Robo2* splicing defects. The *Robo2* mutation also did not segregate with the kidney phenotype (Supplementary Fig. 1), further indicating *Robo1* is the pathogenic mutation. It is interesting to note *Robo1* has been shown to work with *Slit2* in regulating mammary gland branching morphogenesis^9^ and in lung development^10^.

**Figure 2:**
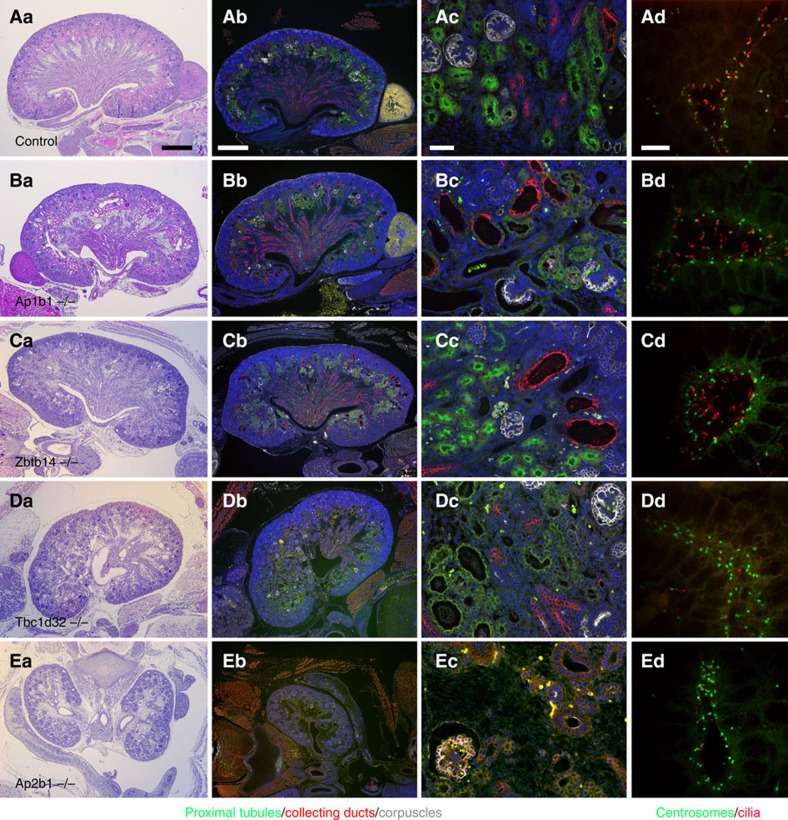
Moderate cystic kidney disease. (**Aa**,**Ba**,**Ca**,**Da**,**Ea**) Haematoxylin and eosin (H&E)-stained sections of kidneys. Scale bar, 500 μm. (**Ab**,**Ac**,**Bb**,**Bc**,**Cb**,**Cc**,**Db**,**Dc**,**Eb**,**Ec**) Kidneys stained with LTA (green), aquaporin-2 (red), T1α (grey) and DAPI (4,6-diamidino-2-phenylindole; blue), to mark proximal tubules, collecting ducts, renal corpuscles and nuclei, respectively. Scale bar, 500 μm (**b**) and 50 μm (**c**). It is noteworthy that Ap1b1 (**B**), Zbtb14 (**C**) and Ap2b1 (**E**) primarily affect the collecting ducts, while Tbc1d32 (**D**) affects the proximal tubules. (**Ad**,**Bd**,**Cd**,**Dd**,**Ed**) Kidneys stained for centrioles (γ-tubulin, green) and cilia (Arl13b, red). Scale bar, 10 μm. It is noteworthy that cilia are normal in Ap1b1 (**B**) and Zbtb14 (**C**) but are short or absent in Tbc1d32 (**D**) and Ap2b1 (**E**). See Supplementary Fig. S2 for more examples.

**Figure 3:**
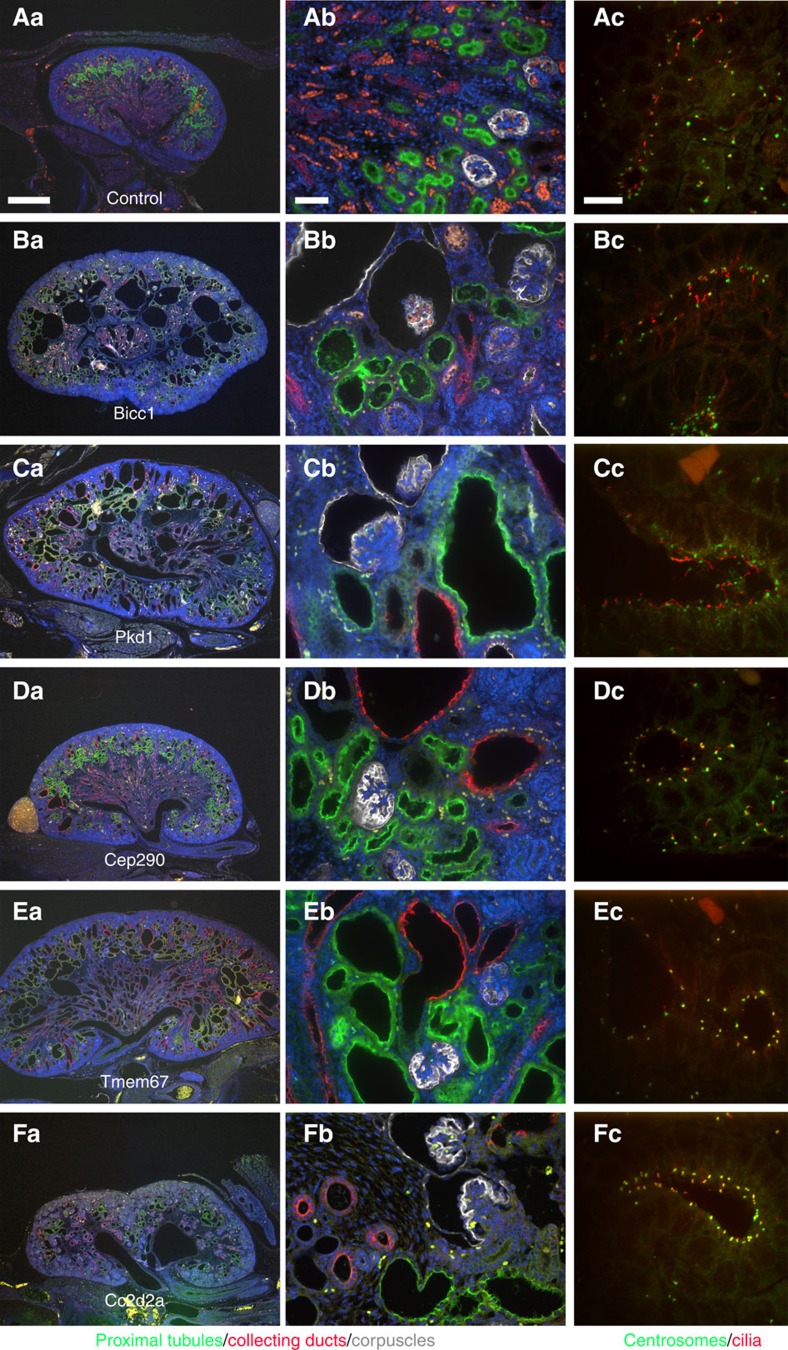
Severe cystic kidney disease. (**Aa**,**Ab**,**Ba**,**Bb**,**Ca**,**Cb**,**Da**,**Db**,**Ea**,**Eb**,**Fa**,**Fb**) Kidneys stained with LTA (green), aquaporin-2 (red), T1α (grey) and DAPI (4,6-diamidino-2-phenylindole; blue), to mark proximal tubules, collecting ducts, renal corpuscles and nuclei, respectively. It is noteworthy that the large cysts in the medulla of the *Bicc1* line (**B**) are positive for T1α, indicating that they are derived from the renal corpuscle, whereas the small cysts/dilations are derived from proximal tubules. The *Pkd1* line (**C**) has dilations and cysts in the renal corpuscles, nephrons and collecting ducts. *Cep290* mutants (**D**) have primarily collecting duct cysts but also show some dilation of the proximal tubules. *Tmem67* lines (**E**) are similar to the *Cep290* presentation, except that the proximal tubule is more affected. Similarly, the *Cc2d2a* line (**F**) shows collecting duct and proximal tubule dilations along with glomerular cysts. The glomerular cysts were observed in individuals without hydronephrosis, suggesting that they are part of the phenotype and not a secondary effect of hydronephrosis. Scale bar, 500 μm (**a**) 50 μm (**b**). (**Ac**,**Bc**,**Cc**,**Dc**,**Ec**,**Fc**) Kidneys stained for centrioles (γ-tubulin, green) and cilia (Arl13b, red). The cilia in Bicc1 (**Bc**) and Pkd1 (**Cc**) mutant kidneys looked similar to wild type and no significant difference was seen in per cent ciliation in these lines. *Cep290* (**Dc**), *Tmem67* (**Ec**) and *Cc2d2a* (**Fc**), all greatly affected ciliation. *Tmem67* and *Cc2d2a* caused a remarkable reduction in per cent ciliation and the cilia that remained were short stubs. *Cep290* was more variable in presentation. It did not have a great effect on per cent ciliation in the ureteric bud tip but did cause a dramatic shortening of the cilia on these cells. Long cilia could be found in other cell types in the kidney. Scale bar, 10 μm.

Cystic kidney disease was commonly observed in the CHD mutants (Fig. 1h–j). The cystic disease identified in our screen could be grouped into three distinct classes. The first group are those with cystic disease that may arise secondary to hydronephrosis. In this class only the kidney with hydronephrosis was cystic, whereas the contralateral kidney or non-hydronephrotic parts of a duplex kidney were not cystic (Fig. 1h, *Nek8*). The second class encompassing moderate cystic disease is marked by a few large kidney cysts and is associated with less extensive tubule dilation (Fig. 1i, *Tbc1d32*). This second class was equally distributed among the cilia versus non-cilia mutants, and included mutations in four cilia genes (*Anks6*, *Lrp2*, *Ptk7* and *Tbc1d32*) and four non-cilia genes (*Ap1b1*, *Ap2b1*, *Bmp10* and *Zbtb14*) (Table 1, Fig. 2 and Supplementary Fig. 2). *Anks6* mutations cause nephronophthisis^11^ and a *Tbc1d32* splice mutation was found in a single patient with oral facial digital syndrome and ambiguous genitalia^12^; however, the other genes are not currently known to cause renal–urinary tract anomalies. The third class of severe cystic kidney disease is marked by extensive cysts throughout the kidneys (Fig. 1j, *Tmem67*). Severe cystic disease was identified only in lines with cilia-related mutations including *Bicc1*, *Cc2d2a*, *Tmem67*, *Cep290* and *Pkd1* (Table 1, Supplementary Table 1 and Fig. 3). *Bicc1* mutations are known to cause cystic renal dysplasia^13^, *Pkd1* is the major cause of autosomal dominant PKD in humans^14^, and *Cc2d2a*, *Tmem67* and *Cep290* are well-established human ciliopathy genes^15^. These severe cystic kidney phenotypes are fully penetrant (Table 1). We also observed kidney phenotypes comprising bilateral and unilateral renal agenesis and/or hypoplasia (Fig. 1f, *Frem2* and 1G, *Plxnd1*). This has variable penetrance and was observed in association with mutations in four non-cilia-related genes: *Frem2*, *Lama5*, *Plxnd1* and *Qrich1* (Table 1). *Frem2* mutations in humans cause Frasier syndrome, which includes renal agenesis and other renal–urinary tract malformations in combination with other organ defects^16^. The other three genes are not known to cause renal–urinary tract malformations in humans.

**Figure 4:**
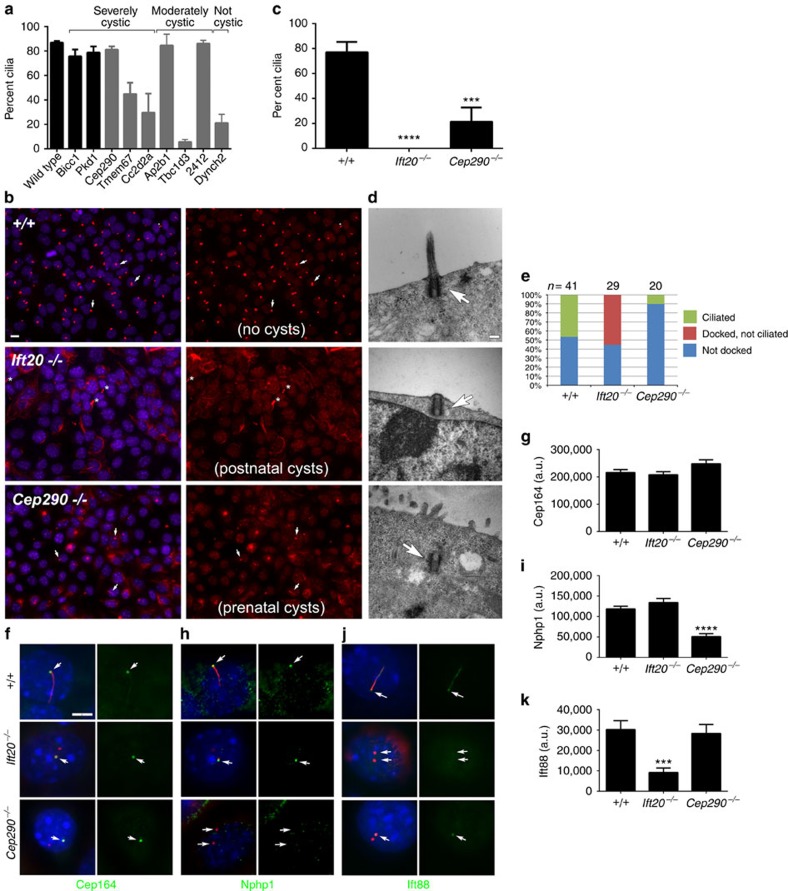
Analysis of centriolar docking in kidney epithelial cells. (**a**) No correlation between cilia and cysts in the prenatal period. Per cent of ciliated centrosomes were measured in developing cortical collecting ducts in wild type and mutants (*N*=3 individuals per line and >100 centrosomes per animal). Dark bars indicate that cilia length was normal, while grey bars indicate that cilia were shorter than normal. (**b**,**c**) Per cent ciliation in kidney epithelial (MEK) cells grown in culture. Arrows mark examples of cilia; * marks midbodies. Scale bar, 10 μm. *N*=3 experiments, >100 cells counted for each condition in each experiment. ***P=0.003 and ****P<0.0001 with respect to wild type. (**d**,**e**) Location of centrioles in cells. MEK cells were grown in culture, serum starved and processed for EM. Arrows mark examples of ciliated (+/+, top row), centriole docked but not ciliated (*Ift20*−/−, middle row) and centriole not docked (*Cep290*−/−, bottom row). (**e**) Distribution of the three classes in the three lines. It is noteworthy that these are random section EMs and the undocked centrioles in wild type and *Ift20* mutants are likely to be the daughter centrioles. Scale bar, 0.2 μm. Centriolar levels of Cep164 (green, (**f**,**g**)), Nphp1 (green, (**h**,**i**)) and Ift88 (green (**j**,**k**)) in the three MEK lines. Red is a combination of anti-acetylated tubulin (6-11B-1) and anti-γ-tubulin (GTU88) label, and blue is DAPI (4,6-diamidino-2-phenylindole). Scale bar, 5 μm. Centriolar levels were quantitated from >25 cells per condition. It is noteworthy that the distal appendage marker Cep164 is not affected by mutations in either *Ift20* or *Cep290*, while the IFT marker Ift88 is affected by the *Ift20* mutation and the transition zone marker Nphp1 is affected by the *Cep290* mutation but not the *Ift20* mutation. ***P=0.0005 with respect to wild type and *P*=0.0016 with respect to Cep290, ****P<0.0001 with respect to wild type or other mutant.

We note the finding of renal anomalies in the CHD mutants is not simply a reflection of the high prevalence of cilia mutations, as 58% of the CHD mutant lines with renal anomalies have mutations in non-cilia genes. Although the severe cystic kidney disease phenotype was exclusively associated with cilia mutations, there was no correlation between ciliary assembly defects and cystogenesis in the prenatal period (Fig. 4a). For example, mutation of the retrograde intraflagellar transport motor, *Dync2h1*, disrupted ciliogenesis but this did not result in the formation of cysts in kidneys in mutants collected at term (Supplementary Fig. 3). Although unexpected, this finding is consistent with our prior observation that prenatal deletion of *Ift20* or *Ift140* did not cause cysts until the postnatal period^17^,^18^. These findings suggest the cilia components *Cep290*, *Cc2d2a* and *Tmem67* may cause prenatal cysts by a fundamentally different mechanism than the cysts caused by intraflagellar transport (IFT) mutations.

### Prenatal cysts and centriolar docking defects

Cep290, Cc2d2a and Tmem67, along with Mks1, Tmem216, B9D1, Tctn1 and Tctn2 form the tectonic complex in the ciliary transition zone^19^. Previously published work shows that *Mks1* and *B9D1* mutants, like *Cep290*, *Cc2d2a*, and *Tmem67* mutants identified here, also exhibit prenatal renal cysts^20^,^21^. It is striking that five components of this complex cause prenatal cysts, as the number of published mutant mice with this phenotype is limited. Work in *Caenorhabditis elegans* suggests the tectonic complex is required for docking of centrioles on the ciliary vesicle and fusion of the nascent cilium to the plasma membrane. In contrast, the IFT proteins appear essential only for ciliary axonemal extension^22^. This suggests that prenatal cyst formation arises from a centriole docking defect rather than a ciliogenesis defect. To examine this possibility, we carried out transmission electron microscopy to assess centriole docking in kidney epithelial cells from *Cep290* and *Ift20* mutants and wild-type cells (Fig. 4d,e). In wild-type and *Ift20* mutant cells we found, as expected, approximately half of the centrioles (presumably the mother centrioles) were docked on the membrane, while the other half (probably daughter centrioles) were in the cytoplasm away from the membrane. In contrast, only 10% of *Cep290* centrioles were docked. We further used immunofluorescence microscopy to assess the distribution of the distal appendage marker Cep164, which should be assembled before ciliary docking and the transition zone marker Nphp1, which should be assembled after docking. This analysis showed that in *Cep290* mutants, assembly of the distal appendages were not affected but formation of the transition zone was blocked. In contrast, the *Ift20* mutation did not affect either of these events (Fig. 4f–k), supporting a role for this protein after docking of centrioles on the membrane. Together, these findings suggest that the disruption of centriole docking plays a critical role in formation of prenatal cysts.

### Human CHD patients have associated renal anomalies

**Figure 5:**
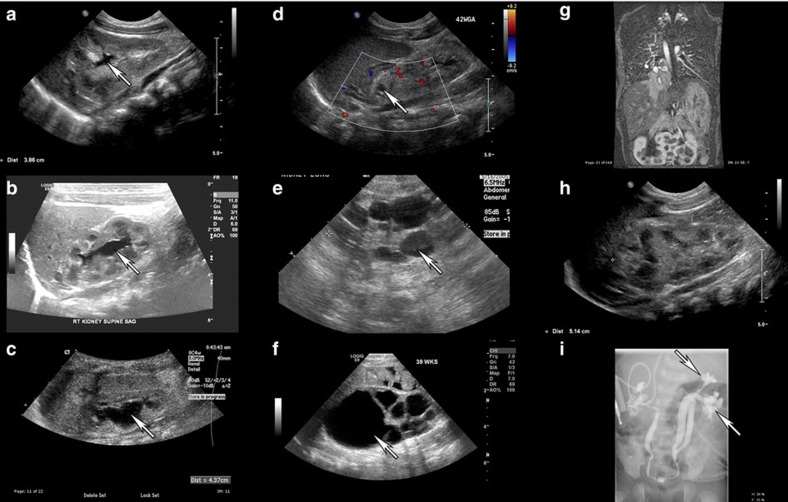
Renal ultrasound of CHD patients demonstrates a spectrum of renal abnormalities. Intrarenal collecting system dilation (arrows) ranging from mild (**a**) to moderate (**b**), to severe (**c**) was observed. (**d**–**f**) Cystic dysplastic abnormalities including an isolated cystic lesion in the upper pole of the kidney (**d**, arrow), multiple cystic lesions throughout the periphery of the kidney parenchyma (**e**, arrow) and cystic dysplastic kidney (**f**, arrow) were observed. Other renal abnormalities including horseshoe kidney (**g**) and duplicated collecting system depicted with renal ultrasound (**h**) and voiding cystourethrogram (**i**) were observed.

To determine the possible clinical relevance of our findings in mouse models, we recruited 77 patients with CHD requiring surgical intervention from the Children's Hospital of Pittsburgh. Retrospective chart review showed 23 (30%) of the CHD patients had renal defects that spanned a spectrum of renal abnormalities (Fig. 5) including renal cysts, kidney agenesis, hydronephrosis and pelvicaliectasis (mild form of hydronephrosis), vesicoureteral reflux, duplicated collecting duct systems (duplex kidneys), ectopic kidney (malpositioned) and horseshoe kidneys (Supplementary Table 3). There was no correlation between cardiac lesion type and the different renal anomalies, similar to the findings in the CHD mutant mice. Together, these findings support a common aetiology for CHD and renal anomalies in the human population.

## Discussion

Our systematic analysis of mouse mutants with CHD showed that many of the mutations causing heart defects also caused renal anomalies. This included a number of genes that had an impact on ciliogenesis. Cilia are microtubule-based organelles that project from the cell surface and can be motile or non-motile, and both types play important roles in development, health and disease^23^. During development, motile and non-motile cilia regulate the specification of left–right patterning. Left–right patterning is critical to the development of the heart, lungs and other organs, and controls the differential placement of the left and right kidneys along the posterior–anterior axis. Non-motile primary cilia, which have important roles in sensory and cell signalling functions, also play critical roles during development. In the kidney, primary cilia defects are strongly associated with cystic kidney disease^24^. The major human autosomal dominant and autosomal recessive PKD proteins localize to the cilia and many genes that affect ciliary structure lead to cystic kidney disease. The mechanism of ciliary action in maintaining tubule architecture and preventing cyst formation is unknown, but various models propose a role for cilia in regulating Wnt signalling or detection of luminal flow to monitor tubule diameter.

**Figure 6:**
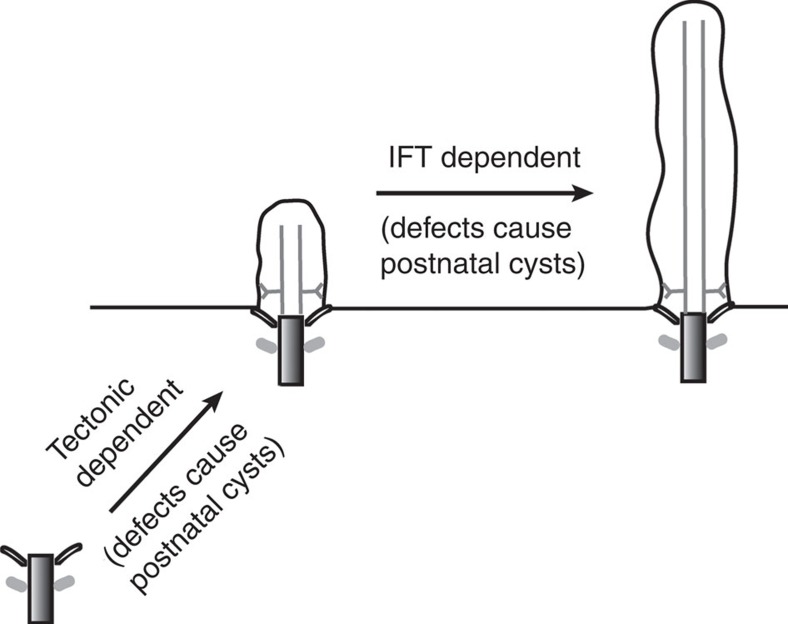
Model to explain the difference between cilia mutations that cause prenatal cysts versus those that only cause cysts in the postnatal period. Defects in the tectonic complex cause prenatal cysts, while defects in IFT components do not cause cysts until after birth. The tectonic complex is thought to be required for ciliogenesis at a step before the docking of the centriole onto the plasma membrane, whereas the IFT complex is not thought to be needed until after docking occurs. This suggests that in the prenatal period a docked centriole is sufficient to provide cues to maintain tubule architecture but in the postnatal period a cilium is required to provide this information.

Curiously, only a subset of cilia mutations caused severe cystic disease in the prenatal period. The fact that cilia are present on the tubular epithelium during prenatal development raises the question of why only a subset of genes affecting the formation of these epithelial cilia cause cysts before birth. To address this question, we examined the placement and maturation of centrioles in the mutants that cause prenatal cysts versus those that do not cause cysts during this time. We found that a prenatal cyst forming *Cep290* mutation blocks ciliogenesis before assembly of the transition zone and plasma membrane docking, whereas an *Ift20* mutation, which does not develop cysts in the prenatal period, did not block ciliogenesis until after the centriole docked and the transition zone was at least partially formed. These findings are consistent with prior studies showing disruption of transition zone assembly in *Cc2d2a* mutant fibroblasts^25^ and failure of centrosomes to migrate to the cell surface in *Cep290* knockdown cells^26^. Taken together, these findings suggest a model where the cilium is not required for maintaining tubule architecture in the prenatal period as long as the centriole is properly docked on the plasma membrane. In the postnatal period, both docked and ciliated centrioles are required for maintaining tubule architecture and preventing cyst formation (Fig. 6). Previous work on mechanisms of tubulogenesis indicated that during the prenatal period, tubules elongate by a convergent extension mechanism, whereas postnatally this occurs via oriented cell division^27^. It is possible that the differences in the requirements of cilia and centrosomes during the two periods of development may reflect this transition. Our model would predict that any gene that prevents ciliation at a step before centriole docking should cause prenatal cysts, such as genes required for centriole biogenesis or formation of transition fibres. A number of mouse models have been generated with defects in centriole biogenesis but null alleles of all of these are lethal before the formation of the metanephric kidney^28^,^29^,^30^,^31^,^32^,^33^ and no studies with floxed or hypomorphic alleles^30^,^34^,^35^ have yet been performed that would independently test our model. It should be noted that our model does not predict that all genes that cause prenatal cysts are required for docking centrioles. Genes such as *Pkd1* and *Bicc1* identified in this screen as causing prenatal cysts are unlikely to function in centriole docking, but may play a role in signalling downstream of ciliary assembly.

The association of CHD with renal anomalies is not restricted to mice, as we also found a high incidence of structural renal defects in CHD patients recruited from the Children's Hospital of Pittsburgh. These CHD patients had renal anomalies across the CAKUT and cystic kidney disease spectrum, similar to what we observed in the CHD mutant mice. Furthermore, our findings are in agreement with a previous epidemiological study in the Atlanta metro area, which showed 23% of ∼8,000 subjects with CHD also had renal abnormalities^36^. Thus, both the mouse and human studies indicate significant overlap in the genetic aetiology of CHD and kidney abnormalities. This probably reflects the conservation of developmental pathways and cell signalling mechanisms that regulate cardiovascular and renal development, including a central role for cilia in the pathogenesis of CHD and renal birth defects. In addition this work indicates that CHD patients would benefit from routine evaluation for renal anomalies to reduce potential renal complications and improve outcome in this high-risk patient population.

Overall, our studies provide compelling evidence of a genetic link between CHD and congenital kidney abnormalities. The finding of renal anomalies in mutants recovered in a CHD screen show the power of forward genetics in yielding novel insights into otherwise seemingly unrelated disease processes. As the genetics of CAKUT and other kidney defects are not well understood, with >90% being of unknown aetiology^37^,^38^, further exploration of this link may yield new insights into kidney anomalies and the mechanism of disease pathogenesis. These findings also suggest the interesting possibility that pooling CHD and CAKUT patient data may improve the power to detect mutations that can contribute to both CHD and renal anomalies. The mutant mouse models generated in this study are curated in the Mouse Genome Informatics Database and cryopreserved at the Jackson Laboratory for open public access. These mutant models will serve as an invaluable resource for exploring the mechanism of both cardiac and renal structural defects.

## Methods

### Mouse breeding

C57Bl/6J mice were mutated with ENU and bred to wild-type animals. Daughters were then bred back to their fathers and their embryos screened for structural birth defects. Details of the breeding and mutation identification are as described^5^.

All mouse work was approved by Institutional Animal Care and Use Committee (IACUCs) at the University of Pittsburgh or the University of Massachusetts Medical School.

### Histology

*Hematoxylin and Eosin*. Paraffin sections for staining were dewaxed with SafeClear (Fisher Diagnostics) and rehydrated with graded aqueous solutions of isopropanol. The sections were stained for 4 min with CAT Hematoxylin (Biocare Medical), rinsed in running tap water for 30 s followed by three quick dips in saturated lithium carbonate and a rinse in distilled water. This was followed by 90% ethanol for 2 min, Edgar Degas Eosin (Biocare Medical) for 2 min and three quick rinses in 100% ethanol. The sections were cleared with Safeclear (two 5-min incubations) and were mounted with Permount (Fisher Scientific).

*Immunofluorescence*. Paraffin sections were dewaxed, rehydrated and subjected to antigen retrieval in an autoclave (250 °F, 40 min) with 10 mM sodium citrate at pH 6. After cooling to ambient temperature, the sections were treated with blocking solution (4% non-immune goat serum, 0.1% Triton X-100, 0.05% SDS and 0.1% fish skin gelatin (Sigma G7765) in TBST (0.05% Tween-20 in Tris-buffered saline pH 7.4)) for 30 min, subsequently washed with TBST and then exposed to primary antibodies overnight at 4 °C. The next day, the sections were washed with TBST, incubated with Alexa Fluor-conjugated secondary antibodies (Life Technologies) for 30 min at room temperature and washed with TBST followed by a rinse with TBS. The antibodies were brought to their working dilutions with 0.1% fish skin gelatin in TBS. The sections were then dipped for 5 s in 4,6-diamidino-2-phenylindole (1 μg ml^−1^ in TBS) and after rinsing with TBS were mounted with Prolong Gold (Life Technologies).

Primary antibodies used in this work include IFT88 (ref. 39), 6-11B-1 (Sigma T6793), GTU-88 (Sigma T5326), T1α (clone 8.1.1, Developmental Studies Hybridoma Bank, University of Iowa), Cep164 (Proteintech 22227-1-AP) and Nphp1 (ref. 40). Lectins used were fluorescein *Dolichos biflorus* agglutinin (Vector Labs FL-1031) as the marker for developing kidney collecting ducts and fluorescein *Lotus tetragonolobus* lectin for kidney proximal tubules (Vector Labs FL-1321).

### Cell culture

Mouse kidney epithelial cells were derived from embryonic day 18 kidneys by dispersing the organs in trypsin, plating on plastic in medium composed of 45% DMEM (4.5 g l^−1^ glucose), 45% F12 and 10% FCS supplemented with penicillin and streptomycin. Epithelial cells were selected by the addition of 150 mM NaCl and 150 mM urea to the medium^41^.

Cells for immunofluorescence staining were grown on glass coverslips until confluent and then serum was reduced to 0.25% for 48 h before fixation and staining^42^,^43^. Quantification of ciliary antigens was done using the measurement tools of Openlab (Perkin Elmer).

Cells for electron microscopy were grown on tissue culture plastic until confluent and then serum was reduced to 0.25% for 48 h before fixation. Cells were fixed by adding 2.5% glutaraldehyde in 0.1 M Na Cacodylate buffer (pH 7.2) to the culture plates one drop at a time until the initial volume of media was doubled. The cells were allowed to stabilize in this solution for 10 min, all the media/glutaraldehyde was removed, fresh 2.5% glutaraldehyde in the same buffer was added and the cells were fixed for an additional 60 min at room temperature. After this primary fixation, the cells were rinsed three times in fresh fixation buffer for 10 min each time and were secondarily fixed with 1.0% osmium tetroxide in H_2_O for 1 h at room temperature. The cells were washed again three times in H_2_O and the cells were dehydrated through a graded series of ethanol (10–100%; three changes), and then transferred to ethanol 100%: SpiPon 812/Araldite 502 resin (50:50/V:V) for 12 h at room temperature. Following resin infiltration, the cells were transferred through three changes of pure SpiPon 812/Araldite 502 epoxy resin each 1 h long. The cells were then changed to a final step of embedding resin mixture and were polymerized for 2 days at 70 °C in their original culture dishes. The plates were then dunked in liquid nitrogen, to separate the bottom of the dish from the cells embedded in the resin. Pieces of the embedded cultures were re-oriented and mounted onto blank stubs with a drop of super glue and the blocks were trimmed and sectioned (100 nm thick). Sections were collected onto 200-mesh copper support grids and contrasted with uranyl acetate and lead citrate, and then imaged using a Philips CM 10 transmission electron microscope, under 80 Kv accelerating voltage. Images were recorded with a Gatan Erlangshen charge-coupled device digital camera.

### Patient recruitment and analysis

Infants <1 year of age with CHD were prospectively enrolled at Children's Hospital of Pittsburgh, University of Pittsburgh Medical Center (Pittsburgh, PA). Informed consent was obtained from parents or guardians of infants. The patient's CHD diagnosis and other developmental abnormality findings were retrieved from the examination of the electronic medical records, with specific focus on information relating to renal and urinary tract abnormalities. This study was conducted in accordance with the human study protocol PRO09090021 approved by the Institutional Review Board of the University of Pittsburgh.

## Additional information

**How to cite this article:** Agustin, J. T. S. *et al*. Genetic link between renal birth defects and congenital heart disease. *Nat. Commun.* 7:11103 doi: 10.1038/ncomms11103 (2016).

## Supplementary Material

Supplementary Information Supplementary Figures 1-3 and Supplementary Tables 1-3

## Figures and Tables

**Table 1:** Renal anomalies associated with genes causing congenital heart defects.

Genes*	**Number of mutants**	**Duplex**	**Hydro-nephrosis**	**Moderate cystic**	**Severe cystic**	**Multiplex**	**Agenesis/hypoplasia**
***Anks6***	**10**	**—**	**—**	**80%**	**—**	**—**	**—**
***Bicc1***	**8**	**—**	**—**	**—**	**100%**	**—**	**—**
***Cc2d2a***	**5**	**—**	**—**	**—**	**100%**	**—**	**—**
***Cep110***	**5**	**—**	**60%**	**—**	**—**	**—**	**—**
***Cep290***	**13**	**15%**	**—**	**—**	**100%**	**—**	**—**
***Dyc2h1***	**6**	**50%**	**—**	**—**	**—**	**—**	**—**
***Lrp2***	**3**	**—**	**100%**	**100%**	**—**	**—**	**—**
***Pkd1***	**4**	**—**	**—**	**—**	**100%**	**—**	**—**
***Ptk7***	**3**	**—**	**—**	**100%**	**—**	**—**	**—**
***Tbc1d32***	**12**	**25%**	**—**	**92%**	**—**	**—**	**—**
***Tmem67***	**12**	**42%**	**—**	**—**	**100%**	**—**	**—**
*Adamts6*	16	—	56%	—	—	—	—
*Ap1b1*	10	—	—	80%	—	—	—
*Ap2b1*	3	—	—	75%	—	—	—
*Bmp10*	2	—	—	100%	—	—	—
*Cxcr4*	5	60%	—	—	—	—	—
*Frem2*	10	—	—	—	—	—	100%
*Lama5*	5	—	—	—	—	—	60%
*Plxnd1*	3	—	—	—	—	—	63%
*Prdm1*	8	38%	—	—	—	—	—
*Qrich1*	3	—	—	—	—	—	67%
*Robo1*	5	—	—	—	—	100%	—
*Slit2*	7	—	—	—	—	100%	—
*Snx17*	3	66%	—	—	—	—	—
*Wnt5a*	7	43%	—	—	—	—	—
*Zbtb14*	4	—	—	100%	—	—	—

^*^Genes and values in bold are ciliary components based on proteomic and other studies. Supplementary Table 1 provides more details on the lines and cardiac phenotypes.
