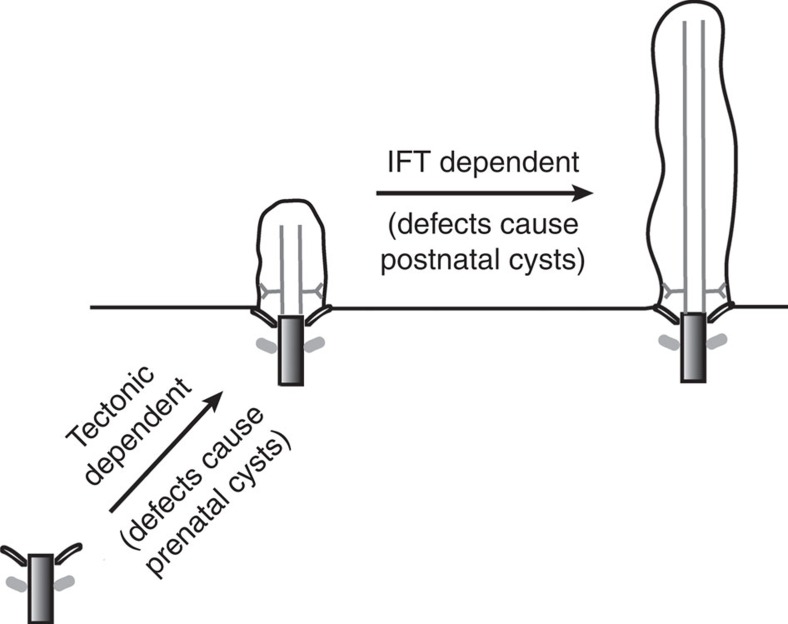# Erratum: Genetic link between renal birth defects and congenital heart disease

**DOI:** 10.1038/ncomms11910

**Published:** 2016-06-08

**Authors:** Jovenal T. San Agustin, Nikolai Klena, Kristi Granath, Ashok Panigrahy, Eileen Stewart, William Devine, Lara Strittmatter, Julie A. Jonassen, Xiaoqin Liu, Cecilia W. Lo, Gregory J. Pazour

Nature Communications 7 Article number:1110310.1038/ncomms11103 (2016); Published 03222016; Updated 06082016

In Fig. 6 of this Article, defects in the tectonic complex were inadvertently labelled during the production process as causing postnatal, rather than prenatal cysts. The correct version of the figure appears below.

## Figures and Tables

**Figure 6:**