# Supplementary material for: Genetic link between renal birth defects and congenital heart disease
Source: Nat Commun. 2016 Mar 22;7:11103. doi: 10.1038/ncomms11103 (PMC4804176; doi:10.1038/ncomms11103)
Supplement: Supplementary Information — Supplementary Figures 1-3 and Supplementary Tables 1-3 [file ncomms11103-s1.pdf]

A

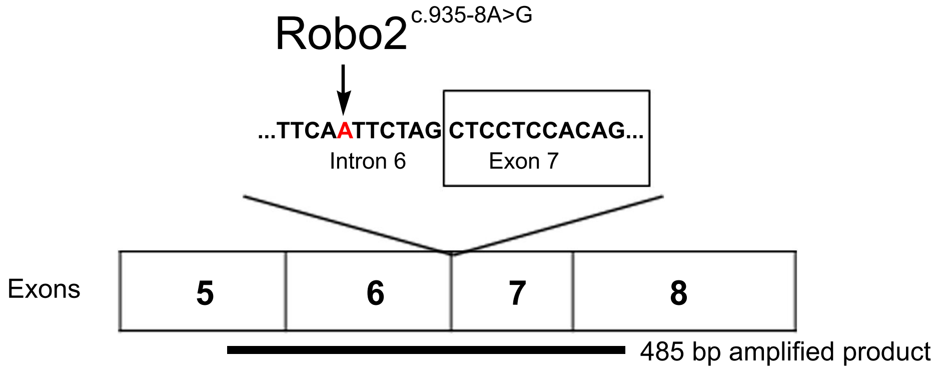

B

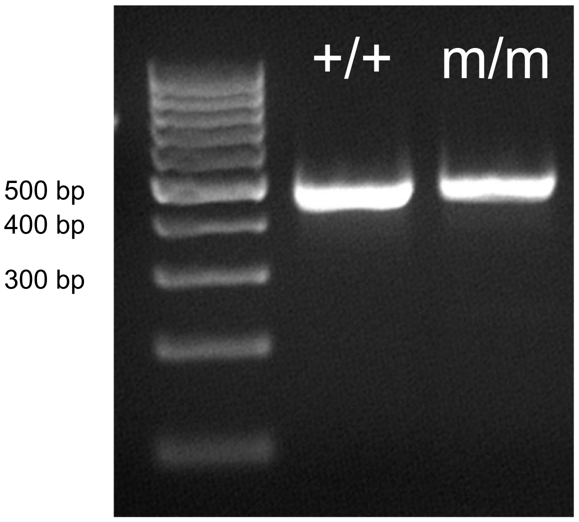

C

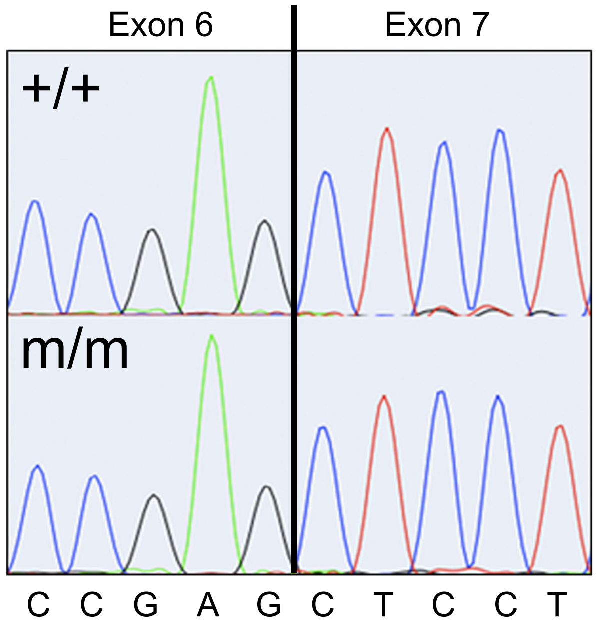

D

Animal	Phenotype	Robo1 Genotype	Robo2 Genotype
872-004-LA	Bilateral Multiplex Kidneys	m/m	+/m
872-004-LB	Bilateral Multiplex Kidneys	m/m	m/m
872-004-NA	Bilateral Multiplex Kidneys	m/m	m/m
872-004-NC	Bilateral Multiplex Kidneys	m/m	m/m
872-004-NE	Bilateral Multiplex Kidneys	m/m	m/m

E

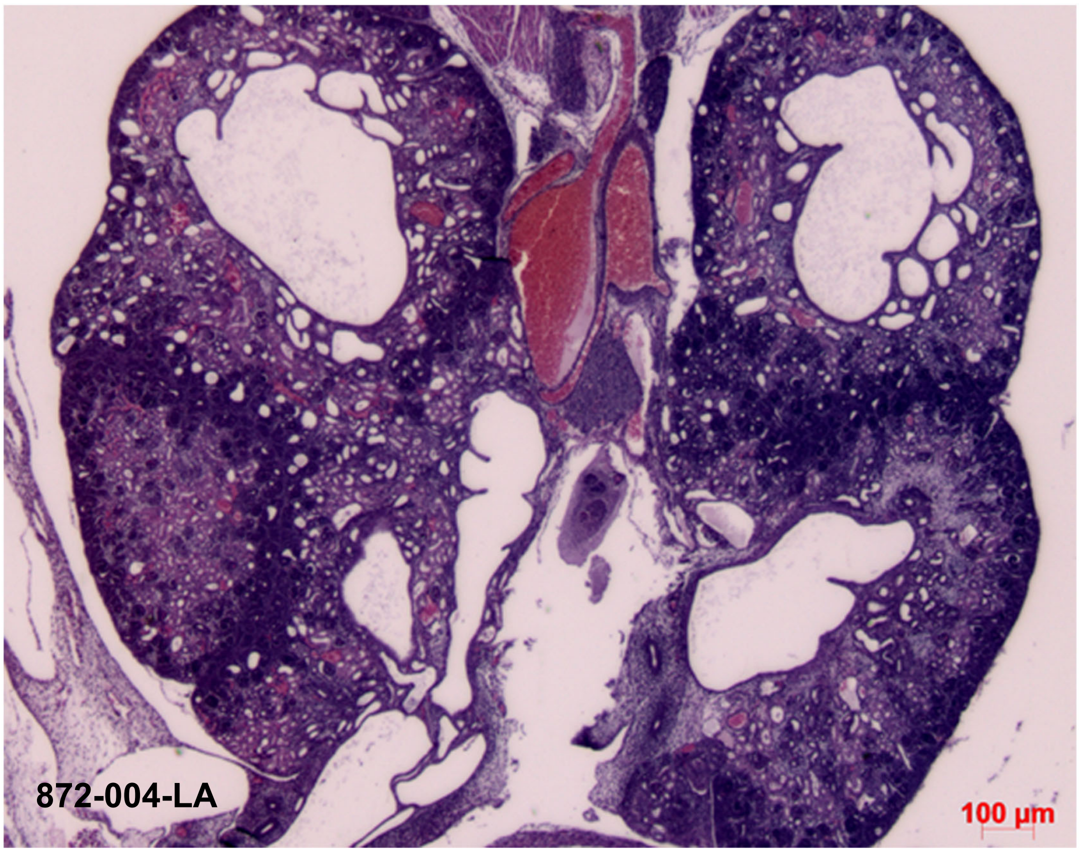

Figure S1

Supplemental Fig. 1: *Robo1* is the likely causal mutation in line 872.

A. Family 872 segregates a T809C transition that causes an I270T missense mutation within the coding region of *Robo1* and an A to G nucleotide substitution near the intron 6-exon 7 splice junction of *Robo2*.

B,C. The *Robo2* mutation does not affect splicing of *Robo2* RNA in skin.

D,E. Segregation analysis shows that animal 872-004-LA is heterozygous for the *Robo2* mutation and this animal shows the multiplex kidney phenotype (**E**).

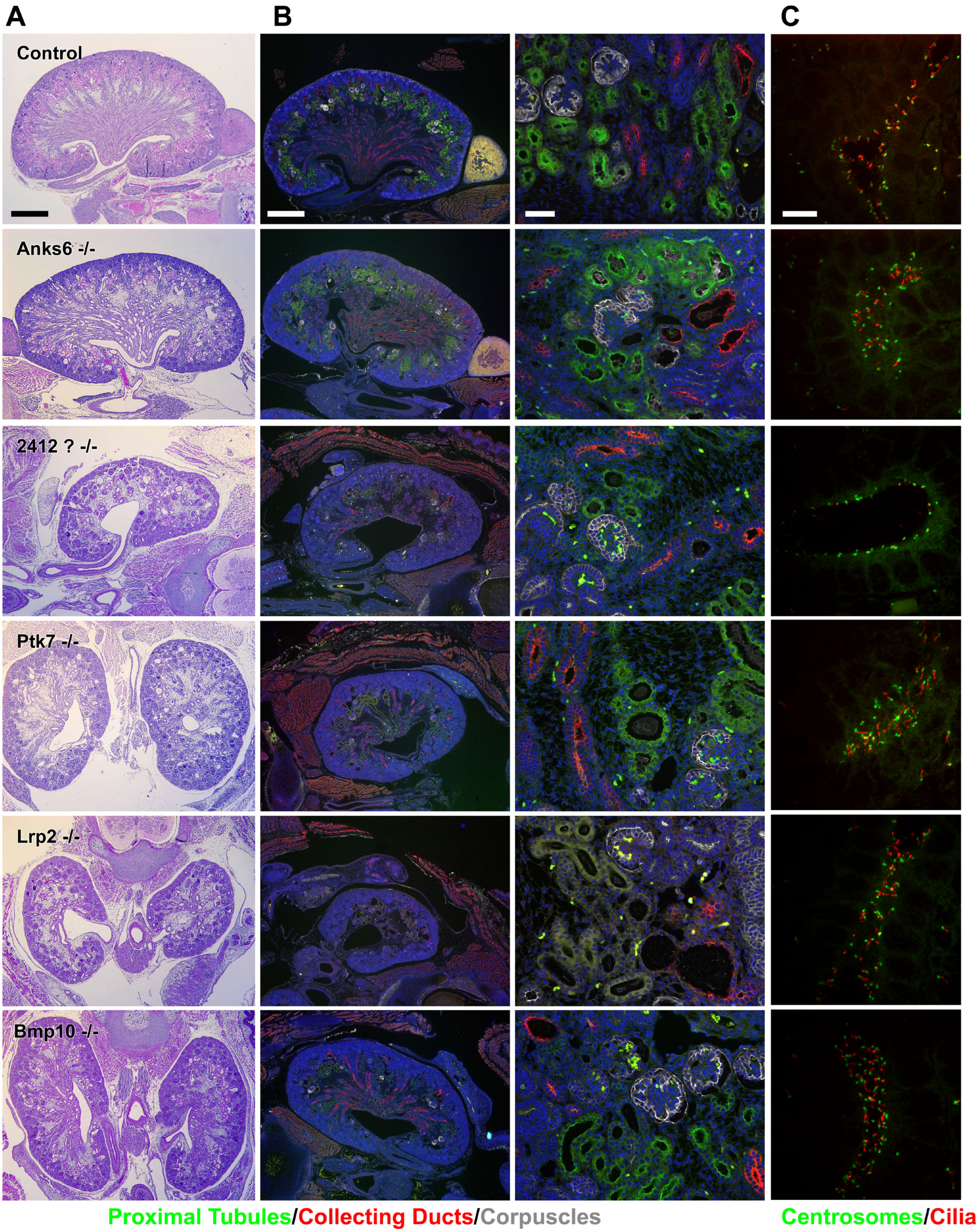
A
B
C
Control
Anks6 -/-
2412 ? -/-
Ptk7 -/-
Lrp2 -/-
Bmp10 -/-
Proximal Tubules/Collecting Ducts/Corpuscles
Centrosomes/Cilia

Figure S2

Supplemental Fig. 2: Moderate cystic kidney disease.

A. H&E stained sections of kidneys. Note small cysts or tubular dilations in all of the mutant lines. Line 2412, *Ptk7*, *Lrp2* and *Bmp10* show hydronephrosis of varying severity.

B. Kidneys stained with LTA (green), aquaporin-2 (red), T1α (grey) and DAPI (blue) to mark proximal tubules, collecting ducts, renal corpuscles and nuclei respectively. Scale bar in left panel is 500 microns and in the right panel is 50 microns. Note that Anks6 and Ptk7 mostly affect the proximal tubule while *Lrp2*, *Bmp10* and line 2412 cause small cysts or dilations in both proximal tubules and collecting ducts.

C. Kidneys stained for centrioles (gamma tubulin, green) and cilia (Arl13b, red). Scale bar is 10 microns. 2412 contains an *Ephb4* mutation that causes cardiac malformations but this mutation does not segregate with the mild cystic or ciliary defect phenotypes. Note that cilia are normal in *Anks6*, *Ptk7*, *Lrp2* and *Bmp10* but are short in line 2412.

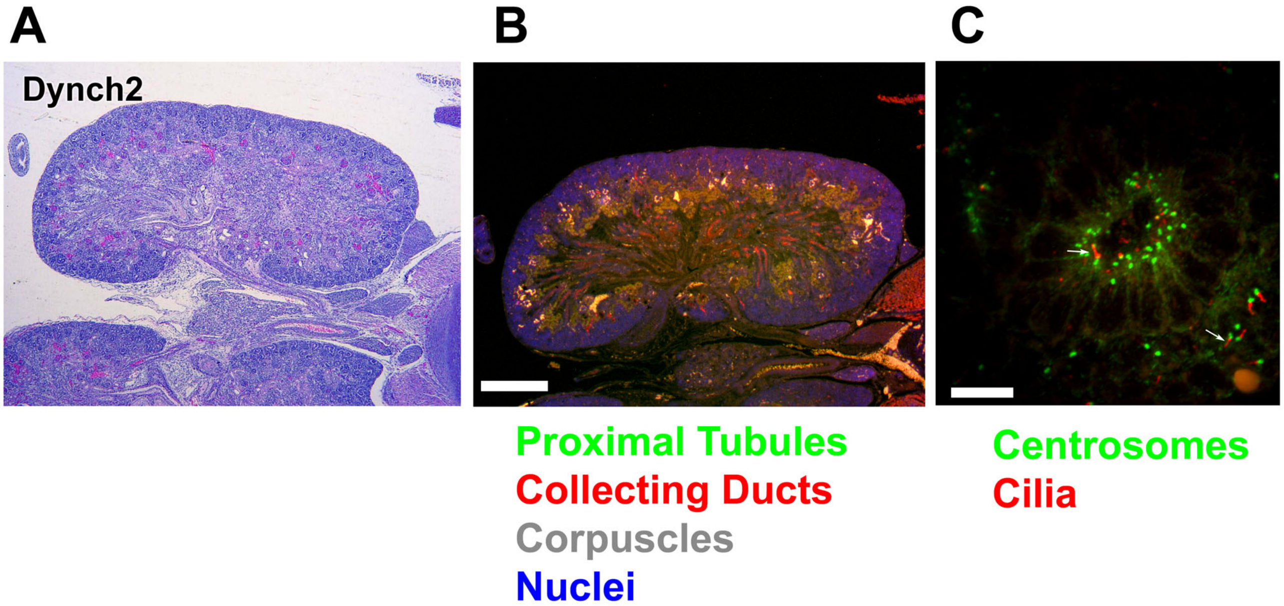
A
Dynch2
B
C
Proximal Tubules
Collecting Ducts
Corpuscles
Nuclei
Centrosomes
Cilia

Figure S3

Supplemental Fig. 3: An IFT Dynein *Dynch2* mutation affects ciliogenesis but does not cause prenatal cysts.

A. H&E stained sections of mutant kidneys. Note that the *Dynch2* kidney is normal.

B. Mutant kidney stained with LTA (green), aquaporin-2 (red), T1α (grey) and DAPI (blue) to mark proximal tubules, collecting ducts, renal corpuscles and nuclei respectively. Scale bar is 500 microns.

C. Mutant kidneys stained for centrioles (gamma tubulin, green) and cilia (Arl13b, red). Scale bar is 10 microns. Note that cilia assembly is disrupted. A few of the remaining cilia are marked with arrows.

Table S1: Identified Lines, Genes and Phenotypes

Line	Gene	Penetrance	Cardiac Phenotype
**Duplex Kidneys/Duplicated Collecting Duct Systems**			
1035	***Cc2d2a***[1]	4 of 5 mutants were duplex.	Complex CHD with heterotaxy
1454	***Cep290***	2 of 7 mutants were duplex.	Complex CHD with heterotaxy
220	*Cxcr4*	3 of 5 mutants were duplex.	pmVSD, mVSD, Ao atresia, RAA, hypo arch, double arch
414	***Dync2h1***	3 of 6 mutants were duplex.	CHD with heterotaxy
1765	*Prdm1*	3 of 8 mutants were duplex.	DORV, AVSD, Ao valve atresia, VSD, Ao arch defects
1625A	*Snx17*	2 of 3 mutants were duplex.	DORV/OA, AVSD
3260	***Tbc1d32***	3 of 7 mutants were duplex.	Complex CHD with heterotaxy
1163	***Tmem67***	3 of 10 mutants were duplex.	Complex CHD with heterotaxy
1291	***Tmem67***	2 of 2 mutants were duplex.	Complex CHD with heterotaxy
3077	*Wnt5a*	3 of 7 mutants were duplex.	DORV, PTA, AVSD, VSD
**Hydronephrosis**			
1879	*Adamts6*	3 of 7 mutants were affected.	DORV/OA
2029	*Adamts6*	3 of 6 mutants were affected.	DORV/OA
2182	*Adamts6*	3 of 3 mutants were affected.	DORV/OA
1468	***Cep110***	3 of 5 mutants were affected.	DORV, AVSD, VSD
2671	***Lrp2***	3 of 3 mutants were affected.	PTA, VSD, Ao arch defects
**Bilateral Multiplex Kidneys**			
1200	*Slit2*	7 affected animals identified.	
872	*Robo1*	5 of 5 mutants were affected.	Biventricular hypertrophy, VSD
**Kidney Agenesis/Hypogenesis**			
414	(not *Dync2h1*)[2]	9 individuals were missing one or two kidneys.	CHD with heterotaxy
3323	*Fras1*	1 of 1 mutant showed agenesis	
1562	*Frem2*	10 of 10 mutants were missing both kidneys.	Biventricular hypertrophy
2966	*Lama5*	3 of 5 mutants showed agenesis/hypogenesis.	DORV
553	*Plxnd1*	5 of 10 mutants were missing one or both kidneys.	PTA/DORV, AVSD, RAA
1863	*Plxnd1*	3 of 6 mutants showed agenesis/hypogenesis.	PTA/DORV, AVSD, RAA
3150	*Plxnd1*	2 of 2 mutants were missing one or both kidneys.	DORV, AVSD, Ao valve atresia, VSD, Ao arch defects
2404	*Qrich1*	2 of 3 mutants were hypomorphic.	DORV, AVSD, noncompaction
3462	*Sc5d*	1 of 1 mutant showed agenesis	
**Moderate Cystic Disease**			
1801	***Anks6***	8 of 10 mutants were affected.	Complex CHD with heterotaxy
1660	*Ap1b1*	8 of 10 mutants were affected.	Complex CHD with heterotaxy
2321	*Ap2b1*	3 of 4 mutants were affected.	DORV/Taussig-Bing, AVSD/VSD, Ao arch defects
2711	*Bmp10*	2 of 2 mutants were affected.	DORV
2671	***Lrp2***	3 of 3 mutants were affected. All were hydronephrotic.	PTA, VSD, Ao arch defects
2445	***Ptk7***	3 of 3 mutants were affected.	DORV, VSD, biventricular hypertrophy
2284	***Tbc1d32***	5 of 6 mutants were affected.	Complex CHD with heterotaxy
2596	***Tbc1d32***	1 of 1 mutant had glomerular cysts.	Complex CHD with heterotaxy
3260	***Tbc1d32***	5 of 5 mutants were affected.	Complex CHD with heterotaxy
1982	*Zbtb14*	4 of 4 mutants were affected.	DORV, AVSD, atrioventricular & semilunar valve defects
2412	(not *Ephb4*)[3]	5 affected animals identified. All were hydronephrotic.	
**Severe Cystic Disease**			
222	***Bicc1***	8 of 8 mutants were affected.	Complex CHD with heterotaxy
1035	***Cc2d2a***	5 of 5 mutants were affected.	Complex CHD with heterotaxy
1454	***Cep290***	8 of 8 mutants were affected.	Complex CHD with heterotaxy
1752	***Cep290***	3 of 3 mutants were affected.	Complex CHD with heterotaxy
2059	***Cep290***	3 of 3 mutants were affected.	Complex CHD with heterotaxy
1163	***Tmem67***	10 of 10 mutants were affected.	Complex CHD with heterotaxy
1291	***Tmem67***	2 of 2 mutants were affected.	Complex CHD with heterotaxy
1585	***Pkd1***	4 of 4 mutants were affected.	Biventricular hypertrophy

[1]Gene names in bold are likely to be ciliary components based on proteomic and other studies.
[2]The cardiac phenotype in line 414 is due to a mutation in *Dynch2* but this segregates independent of the agenesis phenotype.
[3]The cardiac phenotype in line 2412 is caused by a mutation in Ephb4 but this mutation segregates independent of the cystic phenotype.

Table S2: Mutations and corresponding protein changes

Line	Gene	Nucleotide Change	Protein Change
1879	*Adamts6*	c.C447G	p.S149R
2029	*Adamts6*	c.C447G	p.S149R
2182	*Adamts6*	c.C447G	p.S149R
1801	*Anks6*	c.T560A	p.M187K
1660	*Ap1b1*	c.T1094C	p.V365A
2321	*Ap2b1*	c.T1343A	p.M448K
222	*Bicc1*	c.606+2T>C	
2711	*Bmp10*	c.A328G	p.N110D
1035	*Cc2d2a*	c.C2845T	p.R949X
1468	*Cep110*	c.357+2T>A	
1752	*Cep290*	c.1189+2T>C	
1454	*Cep290*	c.T4670A	p.L1557X
2059	*Cep290*	c.T4670A	p.L1557X
220	*Cxcr4*	c.G295A	p.D99N
414	*Dync2h1*	c.T701A	p.V234E
3323	*Fras1*	c.C1228T	p.Q410X
1562	*Frem2*	c.A6875G	p.Y2292C
2966	*Lama5*	c.T333A	p.C111X
2671	*Lrp2*	c.T6612A	p.Y2204X
1585	*Pkd1*	c.T11084A	p.I3695N
553	*Plxnd1*	c.A4727G	p.D1576G
1863	*Plxnd1*	c.2691+1G>A	
3150	*Plxnd1*	c.T4445A	p.M1482K
1765	*Prdm1*	c.A1625G	p.D542G
2445	*Ptk7*	c.T1904A	p.I635
2404	*Qrich1*	c.T1823C	p.M608T
872	*Robo1*	c.T809C	p.I270T
3462	*Sc5d*	c.343+4A>G	
1200	*Slit2*	c.1808+6T>T	
1625A	*Snx17*	c.A431G	p.E144G
2284	*Tbc1d32*	c.1071+2T>A	
2596	*Tbc1d32*	c.C3096A	p.Y1032X
3260	*Tbc1d32*	c.3100+1G>A	
1163	*Tmem67*	c.2322+6T>C	
1291	*Tmem67*	c.T1120A	p.Y374N
3077	*Wnt5a*	c.G670T	p.E224X
1982	*Zbtb14*	c.C419A	p.S140X

Table S3: Clinical characteristics of congenital heart disease patients

ID #	Kidney Phenotypes	Cardiac Phenotype*	Other Phenotypes
7042	Abnormally rotated kidney, Pelvicaliectasis	HLHS	
7199	Caliectasis (dilation of the renal calices)	HLHS	Hypothyroidism
7351	Duplicated collecting duct system, Hydronephrosis	HLHS	
7208	Duplicated collecting system, Vesicoureteral reflux	D-TGA	
7319	Ectopic kidney	DORV, subaortic VSD	
7040	Ectopic kidney, vesicoureteral reflux	ASD, VSD	
7194	Horseshoe kidney	TOF, PA	
7035	Hydronephrosis	ASD, VSD	Tethered cord, hypothyroidism
7288	Mild Hydronephrosis	Truncus arteriosus	
7053	Pelvicaliectasis	D-TGA	
7302	Pelvicaliectasis	HLHS	
7306	Pelvicaliectasis	D-TGA	
7334	Pelvicaliectasis	ASD, VSD, PDA, Interrupted IVC	
7389	Pelvicaliectasis	HLHS	
7417	Pelvicaliectasis	VSD/PDA/ventricular hypertrophy	
7419	Pelvicaliectasis	DORV, PA, complete AVSD, supracardiac TAPVR	Heterotaxy, asplenia
7430	Pelvicaliectasis	Unbalanced AVSD, PA, supracardiac TAPVR	Heterotaxy, asplenia
7058	Pelvicaliectasis, Hydroureter	TOF	Tethered cord, exotropia, neurogenic bladder
7336	Pyelectasis (dilation of the renal pelvis)	D-TGA	
7289	Renal size asymmetry, Solitary cyst seen by ultrasound.	HLHS	
7474	Unilateral Agenesis	HLHS	Single testis
7027	Vesicoureteral reflux	D-TGA	Situs inversus totalis, seizures
7438	Vesicoureteral reflux	HLHS	Aspiration, hypospadias

*Definition of Abbreviations: D-TGA = D-transposition of the great arteries; DORV = double outlet right ventricle; ASD = atrial septal defect; VSD = ventricular septal defect; HLHS = hypoplastic left heart syndrome; TOF = Tetralogy of Fallot; AVSD = atrioventricular septal defect; PA = pulmonary atresia; TAPVR = total anomalous pulmonary venous return.
